# A Decrease in Ambient Temperature Induces Post-Mitotic Enlargement of Palisade Cells in North American Lake Cress

**DOI:** 10.1371/journal.pone.0141247

**Published:** 2015-11-16

**Authors:** Rumi Amano, Hokuto Nakayama, Yurika Morohoshi, Yaichi Kawakatsu, Ali Ferjani, Seisuke Kimura

**Affiliations:** 1 Department of Bioresource and Environmental Sciences, Kyoto Sangyo University, Kyoto-City, Kyoto, Japan; 2 Department of Plant Biology, University of California Davis, Davis, California, United States of America; 3 Department of Biology, Tokyo Gakugei University, Koganei-City, Tokyo, Japan; University of Antwerp, BELGIUM

## Abstract

In order to maintain organs and structures at their appropriate sizes, multicellular organisms orchestrate cell proliferation and post-mitotic cell expansion during morphogenesis. Recent studies using Arabidopsis leaves have shown that compensation, which is defined as post-mitotic cell expansion induced by a decrease in the number of cells during lateral organ development, is one example of such orchestration. Some of the basic molecular mechanisms underlying compensation have been revealed by genetic and chimeric analyses. However, to date, compensation had been observed only in mutants, transgenics, and γ-ray–treated plants, and it was unclear whether it occurs in plants under natural conditions. Here, we illustrate that a shift in ambient temperature could induce compensation in *Rorippa aquatica* (Brassicaceae), a semi-aquatic plant found in North America. The results suggest that compensation is a universal phenomenon among angiosperms and that the mechanism underlying compensation is shared, in part, between Arabidopsis and *R*. *aquatica*.

## Introduction

Multicellular organisms have a wide variety of forms. In particular, plant species display an intriguing variety of leaf shapes and sizes [1,2]. Recently, the molecular mechanisms underlying the diversification of leaf shape have been revealed in various model species [3–5]. The molecular mechanisms underlying the regulation of leaf size, which is one component of final leaf form, have mainly been studied in the model plant *Arabidopsis thaliana* (L.) Heynh. (Arabidopsis hereafter). These studies demonstrated that cell proliferation occurs throughout the developing leaf primordia during very early stages and that cell expansion begins after cessation of proliferation. Finally, the cell proliferation zone is restricted at the basal part of the leaf blade to a constant size [6–9]. Together, these studies suggest that the spatiotemporal coordination of cell proliferation and expansion allows leaves to attain an appropriate final size that is characteristic of each species [10]. To date, a number of genes, whose loss or gain of function is associated with changes in cell number, cell size, or both, have been isolated [7,11–14]. Of note, these studies have proposed that leaf size is not simply a function of cell number and size. Interestingly, in several mutants, defects in cell proliferation are known to induce increased post-mitotic cell enlargement. This phenomenon is called compensation [15,16].

Compensation requires regulatory mechanisms that operate post-mitotically to coordinate cell proliferation and expansion. Post-mitotic compensatory cell expansion (CCE) is thought to be unique to plants [17]. To date, this phenomenon had been observed only in plants in which the genes associated with cell proliferation were mutated or in plants that had been subjected to unusual conditions, such as high γ-ray irradiation [11,18]. Transgenic rice (*Oryza sativa*) plants that overexpress genes encoding Kip-related proteins and the petals of *Antirrhinum majus* mutants also showed compensation-like cell enlargement [19–21]. Although these studies suggest that compensation is widespread in angiosperms, it has been unclear whether compensation occurs in plants in their natural environments.

**Fig 1:**
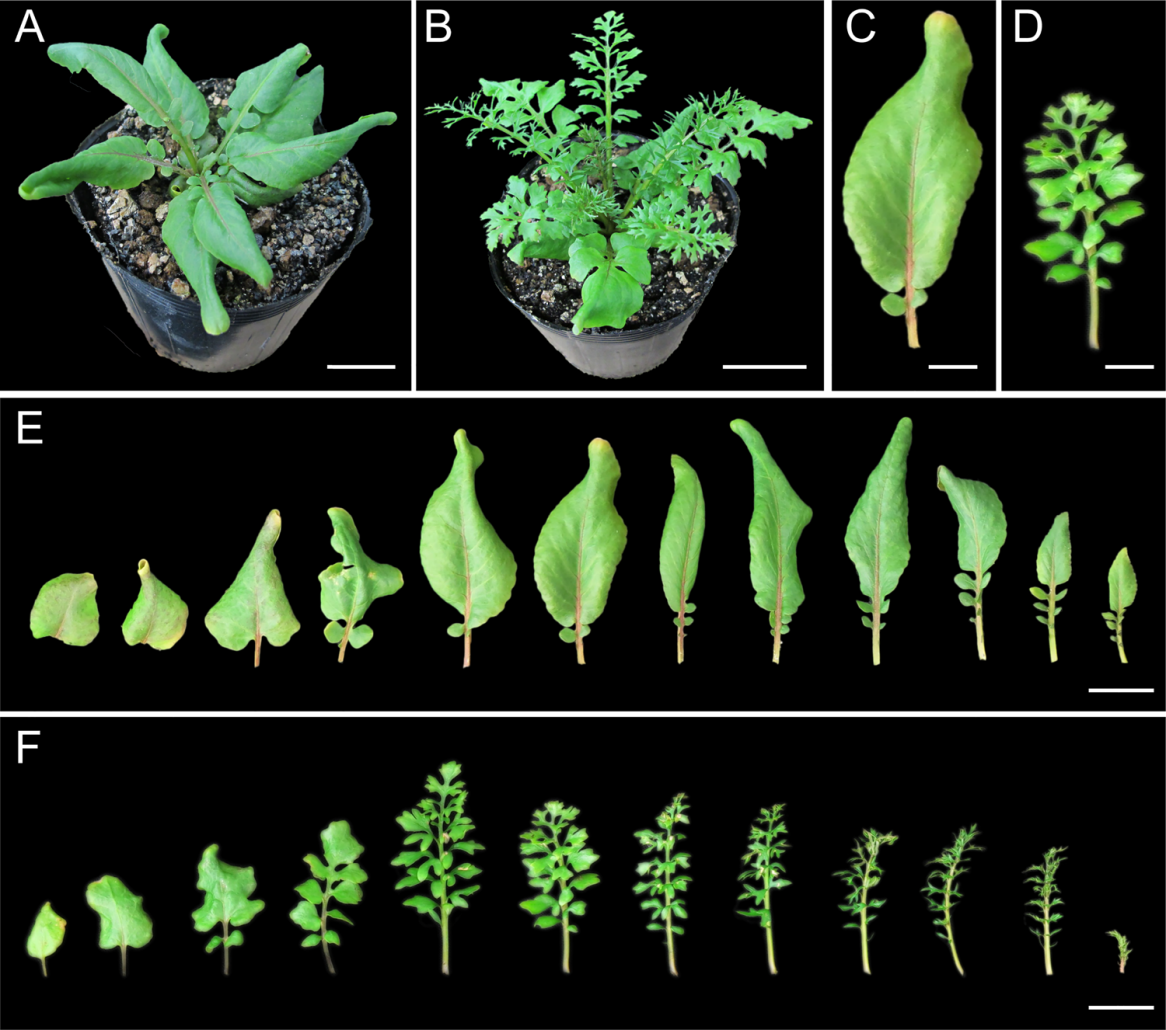
Gross morphology of *Rorippa aquatica* grown at different ambient temperatures. Top views of *R*. *aquatica* plants grown at 30°C (A) and 20°C (B) for 50 days. Leaves (LN6) of *R*. *aquatica* grown at 30°C (C) and 20°C (D). Comparison of leaf forms of *R*. *aquatica* grown at 30°C (E) and 20°C (F). The oldest leaf is depicted at the left and the youngest at the right. Scale bars = 3 cm (A) and (B); 1 cm (C) and (D); 2 cm (E) and (F).

*Rorippa aquatica* (Eaton) EJ Palmer & Steyermark (Brassicaceae), also known as lake cress, is a semi-aquatic plant found in bays, lakes, ponds, and streams in North America [22]. *R*. *aquatica* shows heterophylly, defined as variable leaf form on the same or different shoots of a plant in response to environmental cues. Deeply dissected leaves develop when grown in submerged conditions, whereas simple leaves with smooth margins develop when grown in terrestrial conditions [23,24]. Previously, we showed that the leaf shape of *R*. *aquatica* changes dramatically in response to varying temperatures, as well as to underwater submergence [24]. High ambient temperatures induced formation of leaves with simpler forms compared to those of plants grown at lower temperatures (Fig 1). Previous studies have examined the molecular mechanism underlying heterophylly and have revealed that the regulation of gibberellin (GA) levels via *KNOTTED1-LIKE HOMEOBOX* (*KNOX1*) genes is involved in this phenomenon [25]. In addition, studies using Arabidopsis and *A*. *majus* indicated that compensation may play a role in environmental responses and that it may be induced by changing growth conditions [20,26]. Thus, *R*. *aquatica* showing heterophylly is a useful model to investigate whether compensation occurs in response to environmental cues.

To examine cellular features of *R*. *aquatica* leaves exhibiting heterophylly, we first measured the number and size of palisade cells in the sub-epidermal layer in mature leaves of plants reared at 20°C and 30°C. We then measured the size and complexity of adaxial epidermal cells and investigated in parallel the features of inner tissue cells in mature leaves. Finally, we analyzed expression of *R*. *aquatica* genes whose orthologs in Arabidopsis have been implicated by loss- or gain-of-function mutation analysis in triggering CCE. Together, these results showed that lower temperature (20°C) induces compensation in *R*. *aquatica*. To our knowledge, this is the first report that confirms the occurrence of compensation in response to environmental changes under natural conditions.

## Material and Methods

### Plant materials and growth conditions

*R*. *aquatica* plants were grown in a growth chamber under continuous illumination with light intensity of ~50 μmol photons m^-2^ s^-1^. Seedlings were planted in pots containing soil and watered every 2 days with 1/2 HYPONeX solution (HYPONeX, Japan). For histological observation, plants were cultivated at 20°C or 30°C for 50 days. For RNA extraction, plants were cultivated at 20°C or 30°C for 1 month. Leaf primordia were then frozen in liquid nitrogen immediately after sampling and stored at -80°C until RNA extraction.

### Histological observations

**Fig 2:**
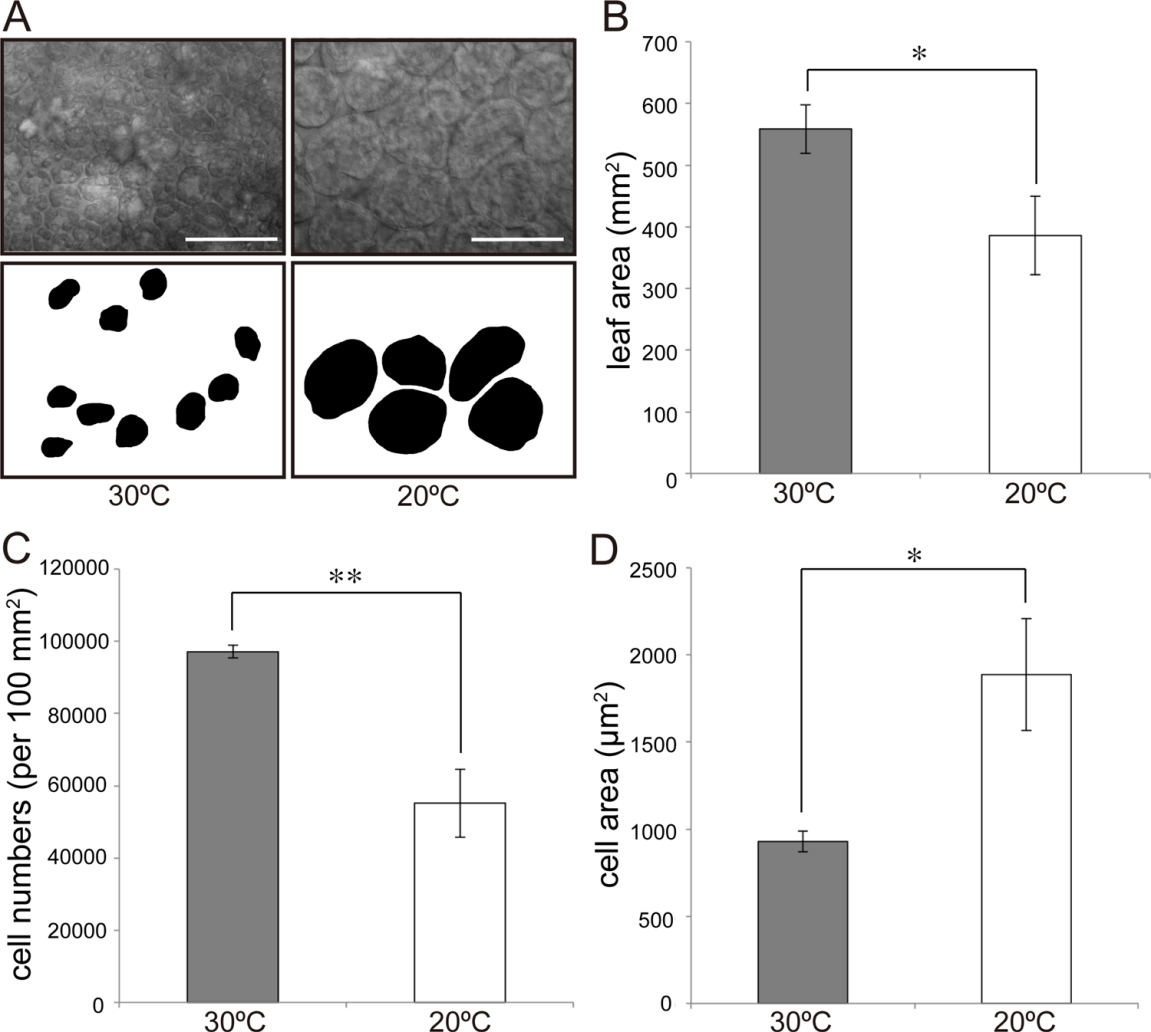
Cellular phenotypes of leaves from plants grown at 30°C and 20°C. (A) Palisade cells in LN6 of *Rorippa aquatica* grown at 30°C (left) and 20°C (right). The upper panels show differential interference microscopy images, and the lower panels show the silhouettes of randomly selected cells. Scale bars = 100 μm. (B–D) Leaf area, number of cells per 100 mm^2^, and palisade cell area, respectively. Error bars represent the standard error (SE); * = *p* < 0.05; ** = *p* < 0.01 by Student’s *t*-test (*n* = 6).

The distal portions of mature leaves (leaf number 6; hereafter LN6) were used for histological observation (*n* = 6 biological replicates) to measure leaf area, cell area, and cell number. In the case of dissected leaves, apical leaflets were used for observations (Fig 1). To measure leaf area, leaves were photographed using a digital camera (PowerShot G11; Canon, Japan). Leaves were fixed in a formalin–acetic acid–alcohol (FAA) solution and cleared using a chloral hydrate solution as described previously [27]. Palisade cells in the sub-epidermal layer were observed using a differential interference microscope (DMI6000; Leica, Germany) and were photographed using a CCD camera (DFC3600 FX; Leica, Germany). Leaf and cell areas were calculated using ImageJ release 1.47v (http://rsb.info.nih.gov/ij/). To calculate cell area, a total of 20 cells in each sample were analyzed (Fig 2A).

### Calculation of complexity in epidermal cells

To calculate the complexity of epidermal cell forms, we used a dental paste to create a mold of the cells (Take 1 Advanced; Kerr, USA). The distal parts of mature leaves (LN6) were used to calculate complexity (*n* = 6 biological replicates). In the case of dissected leaves, apical leaflets were used. Pictures of epidermal cells were traced with Adobe Photoshop CS4 (Adobe, USA), and epidermal cell areas were subsequently calculated. Epidermal cell form complexity was estimated by the dissection index (DI), calculated as $(cell\;periphery)/\sqrt{cell\;area}$. All calculations were performed using ImageJ 1.47v (http://rsb.info.nih.gov/ij/). A total of 20 cells in each sample were analyzed to calculate epidermal cell DI.

### Morphological observations

The central part of the LN6 leaf blade was fixed as previously described. The fixed samples were dehydrated in an ethanol series (50, 60, 70, 80, 90, 95, 99.5, and 100% v/v; 30 min per grade of alcohol) and stored overnight in 100% (v/v) ethanol at room temperature. The samples were embedded in Technovit resin (Technovit 7100; Heraeus Kulzer GmbH, Germany). Embedded samples were sectioned using a microtome (HM325; Thermo Scientific, Japan) and were subsequently stained with 0.1% toluidine blue. The stained sections were observed using an optical microscope (Wraycam G500; Nikon, Japan) and photographed with a CCD camera (Eclipse E200; Nikon, Japan; *n* = 6 biological replicates). Cell area (excluding palisade cells) and leaf thickness were measured using ImageJ 1.47v (http://rsb.info.nih.gov/ij/). A total of 20 cells from three independent locations in the central portion of the leaf blade were analyzed in order to calculate cell area and leaf thickness.

### RNA extraction, cDNA synthesis, and molecular cloning

Total RNA from leaf primordia of plants grown for 1 month was extracted using an RNeasy Plant Mini Kit (Qiagen, USA) including DNase I treatment. cDNA was synthesized from 1 μg of the total RNA using Transcriptor Universal cDNA Master (Roche, USA). Diluted cDNA was used for Real-time PCR. We used de novo assembly data from a previously performed mRNA-seq [25] to design primers for PCR amplification specific to the genes of interest. Accession numbers are LC054303 (Ra *AN3*), LC054304 (Ra *KRP2*), LC068567 (Ra *ERECTA*), LC068568 (Ra *FUGU2*), and LC068569 (Ra *FUGU5*).

### Phylogenetic analyses

Predicted amino acid sequences of cloned genes were aligned using ClustalW and readjusted manually when necessary. Phylogenetic trees were reconstructed with MEGA5 [28] using the neighbor-joining method [29]. Bootstrap values were derived from 1000 replicate runs.

### Real-time PCR analyses

Total RNA was extracted from leaf primordia of plants grown for 1 month and used to synthesize cDNA, as described above. To investigate the expression levels of *ANGUSTIFOLIA3* (*AN3*), *ERECTA*, *FASCIATA1/FUGU2* (*FUGU2*), *AVP1/FUGU5* (*FUGU5*), and *KIP-RELATED PROTEIN2* (*KRP2*) orthologs, expression analysis was conducted using the following gene-specific primer pairs: RaAN3_RT_F and RaAN3_RT_R; RaERECTA_RT_F and RaERECTA_RT_R; RaFUGU2_RT_F and RaFUGU2_RT_R; RaFUGU5_RT_F and RaFUGU5_RT_R; and RaKRP2_RT_F and RaKRP2_RT_R (S1 Table). Real-time PCR amplification was performed using the KAPA SYBR Fast qPCR kit (Kapa Biosystems, USA) in a 7500 Real-Time PCR System (Applied Biosystems, Japan). Experiments were performed in quadruplicate from independent tissue RNA extractions (n = 4 biological replicates) with three technical replicates. Expression was normalized to an *Ra TUB4 ß* control (RaTUB4_RT_F and RaAN3_RT_R; S1 Table).

## Results

### Microscopic observation of leaf palisade cells

To investigate whether cellular changes are induced by different temperatures, cell count and cell area of sub-epidermal palisade tissue in LN6 leaves were compared in plants grown at 30°C and 20°C (Figs 1 and 2). We found that leaf area of plants grown at 30°C was larger than that of plants grown at 20°C (Fig 2B). The number of sub-epidermal palisade cells per 100 mm^2^ at 30°C and 20°C was approximately 97,090 and 55,180, respectively (Fig 2C), and the average cell area was approximately 930 and 1880 μm^2^ at 30°C and 20°C, respectively (Fig 2D). In other words, number and area of sub-epidermal palisade tissue cells in plants grown at 20°C were 49% lower and 57% higher, respectively, than those in plants grown at 30°C. Altogether, our results suggest that CCE is induced in the leaves of *R*. *aquatica* plants grown at 20°C. Incidentally, microscopic observation in a previous study found that palisade cell area of plants reared at 20°C and 25°C was similar [25]; however, the growing conditions differed between that study and the present study. In the previous study, LN7 leaves from plants that had been cultivated for only 30 days were used [25] and were as such younger and less expanded than those used in the present study. We believe that differences in sampling stage and in leaf position used influenced the results of sub-epidermal cell size.

### Observation of epidermal cells and leaf cross-sections

**Fig 3:**
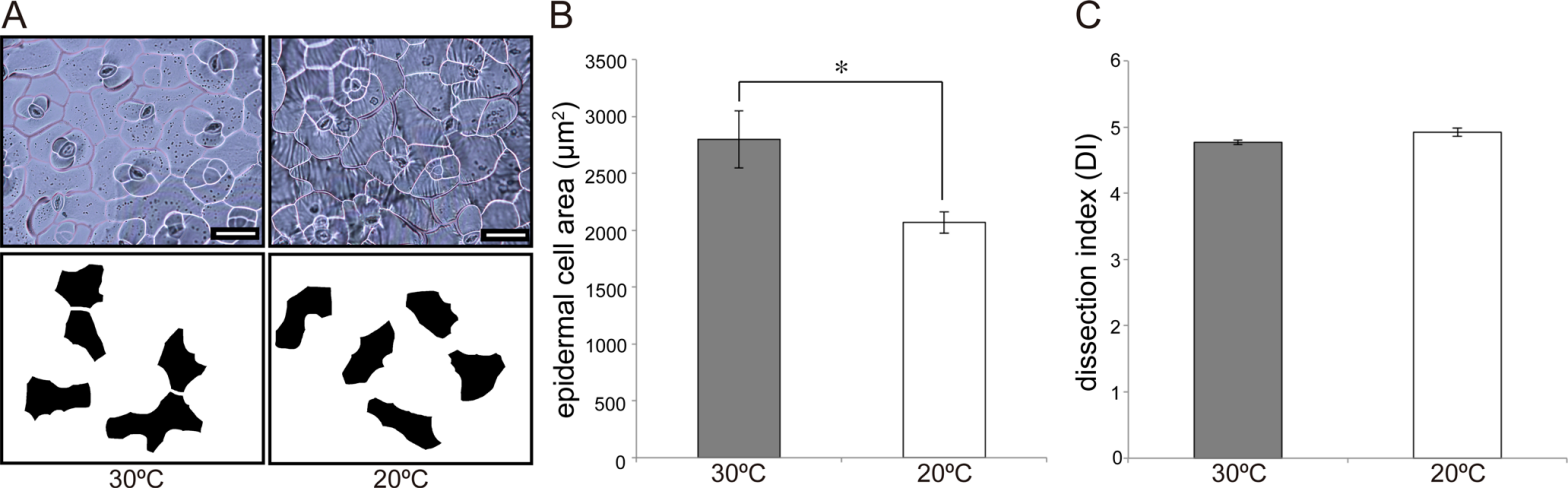
Observation of epidermal cells from plants grown at 30°C and 20°C. (A) Epidermal cells in LN6 of *Rorippa aquatica* grown at 30°C (left) and 20°C (right). The upper panels show images of epidermal cells, and the lower panels show the silhouettes of randomly selected cells. Scale bars = 50 μm. (B) Epidermal cell area. (C) Dissection index (DI) of epidermal cells. Error bars represent the standard error (SE); * = *p* < 0.05 by Student’s *t*-test (*n* = 6).

In addition to the cells in the sub-epidermal layer (palisade tissue), cell area and dissection index (DI) of LN6 adaxial epidermal cells of plants reared at 30°C and 20°C were also analyzed (Fig 3A). Interestingly, whereas epidermal cell area was larger after growth at 30°C than at 20°C (Fig 3B), DI did not significantly differ between plants grown at the two temperatures (Fig 3C).

**Fig 4:**
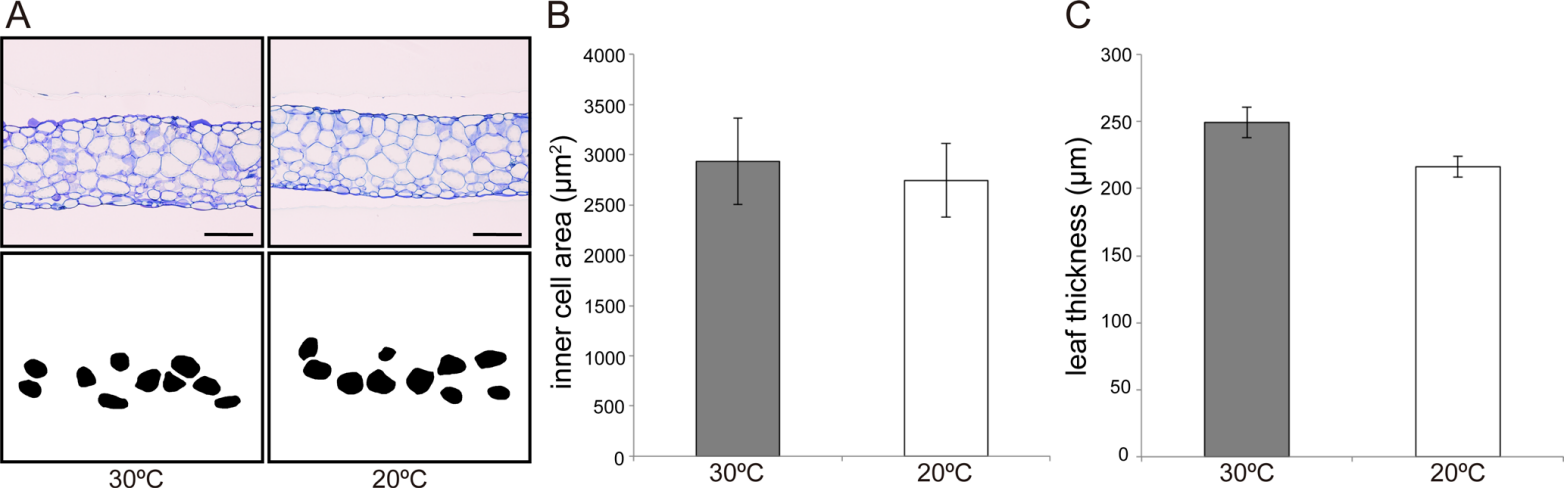
Observation of inner structure of leaves of *Rorippa aquatica* grown at 30°C and 20°C. (A) Inner cells in LN6 leaf blade of *R*. *aquatica* grown at 30°C (left) and 20°C (right). Cross-sections in the upper panels show images of inner leaf tissue cells, and the lower panels show the silhouettes of randomly selected cells. Scale bars = 100 μm. (B) Area of inner cells. (C) Thickness of leaves. Error bars represent the standard error (SE) (*n* = 6).

We also examined cells in inner leaf tissues, excluding palisade and epidermal cells, to determine whether their form and/or size were affected by different ambient temperatures. As shown in leaf cross-sections, cell area of inner tissue was similar between leaves grown at different temperatures (Fig 4A and 4B). Leaf thicknesses between adaxial and abaxial epidermal cells were approximately 215 and 250 μm, respectively, for plants reared at both 30°C and 20°C (Fig 4C).

### Expression analysis of orthologous genes involved in compensation

**Fig 5:**
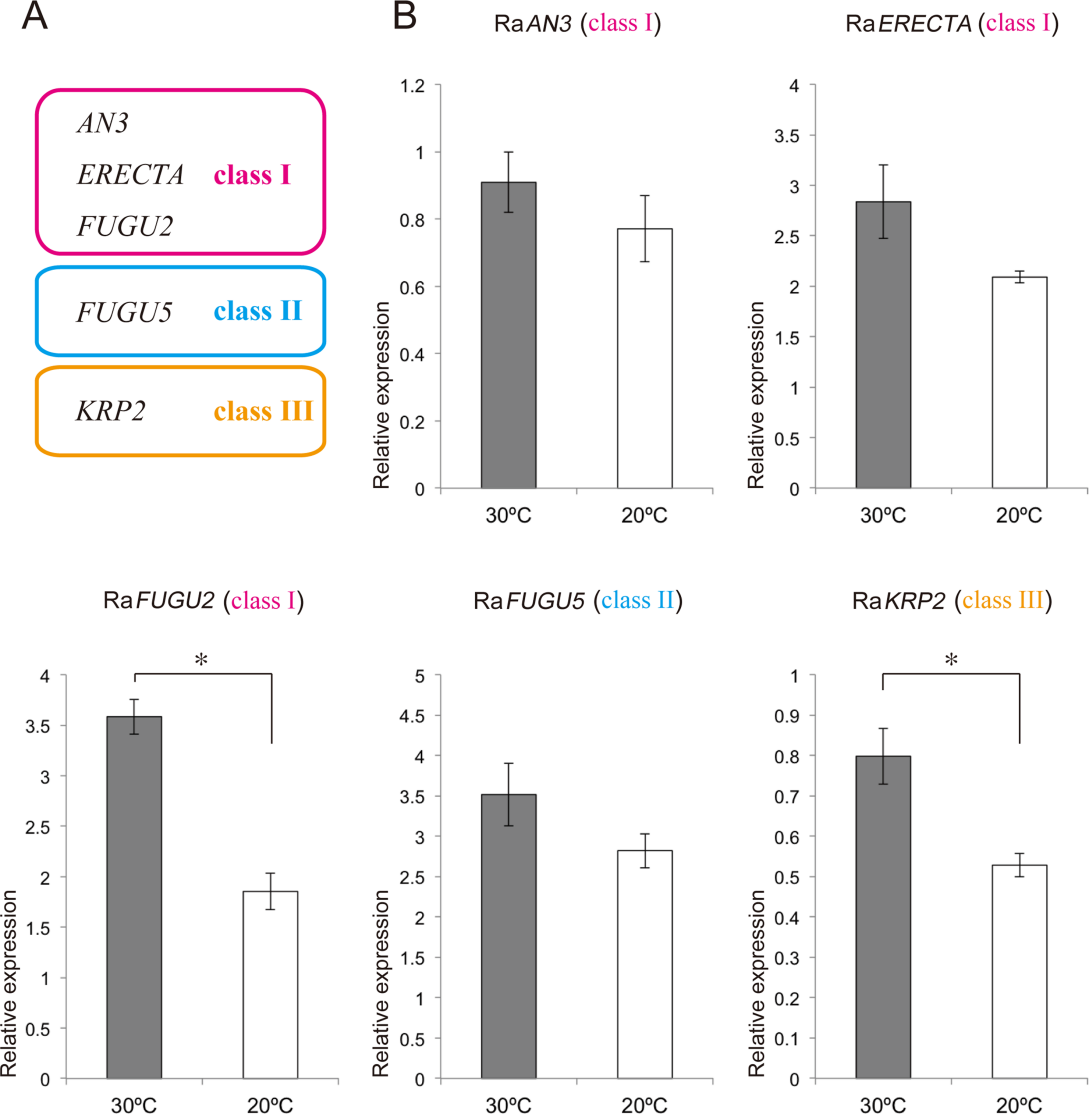
Expression analyses of orthologous genes related to compensation. (A) Schematic presentation of three classes of compensation. (B) Expression levels of Ra *AN3*, Ra *ERECTA*, Ra *FUGU2*, Ra *FUGU5*, and Ra *KRP2* in leaf primordia of *Rorippa aquatica* grown at 30°C and 20°C. Error bars represent the standard error (SE). * = *p* < 0.05 by Welch’s *t-*test (*n* = 4).

To examine the molecular mechanism behind the drastic size increase of *R*. *aquatica* leaf palisade cells, we analyzed expression levels of orthologs of *AN3*, *ERECTA*, *FUGU2*, *FUGU5*, and *KRP2*. The misexpression of each gene is known to trigger different modes of CCE that are sub-classified into three different classes in Arabidopsis (class I: *an3*, *erecta*, and *fugu2*; class II: *fugu5*; class III: *KRP2*o/x) [7,11,19]. These orthologs in *R*. *aquatica* were identified by BLAST searches, which revealed that putative amino acid sequences encoded by the isolated fragments were similar to those of each target gene in Arabidopsis. In addition, multiple sequence alignments showed that each putative protein had a characteristic functional domain or domains conserved among the homologs in a diverse array of species (S1–S5 Figs). Our phylogenetic analyses consistently supported the identity of the orthologs of the targeted genes. Importantly, quantitative RT-PCR showed that although the expression levels of Ra *AN3*, Ra *ERECTA*, and Ra *FUGU5* were unaffected, that of Ra *FUGU2* was significantly reduced in leaf primordia of *R*. *aquatica* grown at 20°C as compared to those of plants grown at 30°C (Fig 5). Thus, the reduction in Ra *FUGU2* expression levels might be associated with the induction of CCE in *R*. *aquatica*. The expression level of Ra *KRP2* was also reduced in the leaf primordia of plants grown at 20°C (Fig 5). However, previous studies using a *KRP2* over-expressor showed that it is the higher expression of *KRP2* that induces CCE [7]. Therefore, the reduced expression of Ra *KRP2* observed in this study might not be related to CCE in *R*. *aquatica*.

## Discussion

### Compensation as a response to change in ambient temperature

In the present study, we demonstrated that CCE is induced in response to environmental factors such as altered ambient temperature. This is the first report that confirms the occurrence of CCE without genetic manipulation and under natural conditions. For example, in plants grown at 20°C, the number of sub-epidermal palisade cells per unit area was significantly lower than that in plants grown at 30°C, whereas sub-epidermal cell size was significantly larger than that in plants grown at 30°C. This cellular behavior seems to be similar to that observed in Arabidopsis mutants exhibiting CCE [7,11–14]. Genetic studies using the *oligocellula* (*oli*) mutant series demonstrated that there is a threshold for triggering compensation [12]. Double mutants such as *oli2-1 oli5-1* and *oli2-1 oli7-1* showed that a large decrease in cell number (e.g., 40–60%) effectively triggers CCE [12]. In *R*. *aquatica*, the sub-epidermal palisade cell density in plants grown at 20°C was approximately 50% lower than in plants grown at 30°C; therefore, a reduction in cell number may suffice to induce compensation. On the other hand, a previous study suggested that it is not the total number of cells in the leaf primordia, but rather the cell proliferation pattern and/or activity that is important for triggering compensation [17]. Indeed, cell proliferation pattern and activity in leaf primordia are altered by changes in ambient temperature in *R*. *aquatica* [25]. Therefore, compensation in *R*. *aquatica* may be induced by such stimuli as were indicated before. Additionally, to reveal whether epidermal and inner cells also exhibit CCE, we examined the features of leaf inner tissue cells using histological cross-sectioning in *R*. *aquatica*. The analysis showed that cell enlargement does not occur in leaf inner cells. Together, our findings indicate the possibility that CCE could preferentially occur in the palisade cells of the sub-epidermal layer in *R*. *aquatica*.

Previous studies revealed that there are three classes of CCE (class I–III) [7,17]. Studies using a *fugu2* mutant showed that the *FUGU2* gene encodes the p150 subunit of Chromatin Assembly Factor 1, and the *fugu2* mutation triggers class I CCE via cell-cycle arrest [14]. Importantly, the results of our quantitative RT-PCR analyses revealed that the expression level of a *FUGU2* ortholog (Ra *FUGU2*) significantly decreased in the leaf primordia of plants grown at 20°C, whereas the expression levels of the *AN3*, *ERECTA*, and *FUGU5* orthologs remained unaffected. This suggests that chromatin structure differs between plants grown at 20°C and 30°C, inducing class I compensation. Moreover, the *fugu2* Arabidopsis mutant develops leaves that are more narrow and serrated than the wild type [7]. Therefore, in addition to the cellular behavior (i.e., occurrence of CCE), the deeply dissected leaves of *R*. *aquatica* grown at 20°C appear somewhat similar to those of *fugu2* mutants. However, further detailed analyses are required to determine whether a shared mechanism exists between morphologically close leaf forms in *R*. *aquatica* and Arabidopsis.

A previous study revealed that chloroplast proliferation is promoted in the enlarged cells that exhibit compensation [30]. Thus, compensation may be a mechanism to secure photosynthetic activity by increasing leaf area and/or thickness and promoting chloroplast proliferation in response to a defect in cell proliferation. If so, aspects of photosynthetic activity, such as the efficiency of CO_2_ fixation per unit of leaf-area, should be investigated in plants exhibiting CCE. However, it is important to note that not only the total number of sub-epidermal cells in leaves, but also leaf morphology differed between *R*. *aquatica* plants grown at 20°C and 30°C. Hence, further analyses are necessary to determine the adaptive significance of compensation, as is the case with its molecular mechanisms.

Finally, our findings provide evidence that compensation is a universal phenomenon seen in nature. Moreover, in *R*. *aquatica* plants grown at a lower temperature, a failure to stably maintain a silent chromatin state via the reduction of Ra *FUGU2* expression (the Arabidopsis counterpart of chromatin assembly factor-1: CAF-1) might trigger a decrease in the number of palisade cells and induce CCE. Further analyses using plants other than Arabidopsis will provide insight into the mechanisms underlying the establishment of appropriate organ size in response to environmental cues.

## Supporting Information

S1 Fig A phylogenetic tree and alignments of *AN3* orthologs. (TIF)

S2 Fig A phylogenetic tree and alignments of *ERECTA* orthologs. (TIF)

S3 Fig A phylogenetic tree and alignments of *FUGU2* orthologs. (TIF)

S4 Fig A phylogenetic tree and alignments of *FUGU5* orthologs. (TIF)

S5 Fig A phylogenetic tree and alignments of *KRP2* orthologs. (TIF)

S1 Table List of oligonucleotide PCR primers used in this study. (XLS)
