# Supplementary figures and images for: A Decrease in Ambient Temperature Induces Post-Mitotic Enlargement of Palisade Cells in North American Lake Cress
Source: PLoS One. 2015 Nov 16;10(11):e0141247. doi: 10.1371/journal.pone.0141247 (PMC4646676; doi:10.1371/journal.pone.0141247)

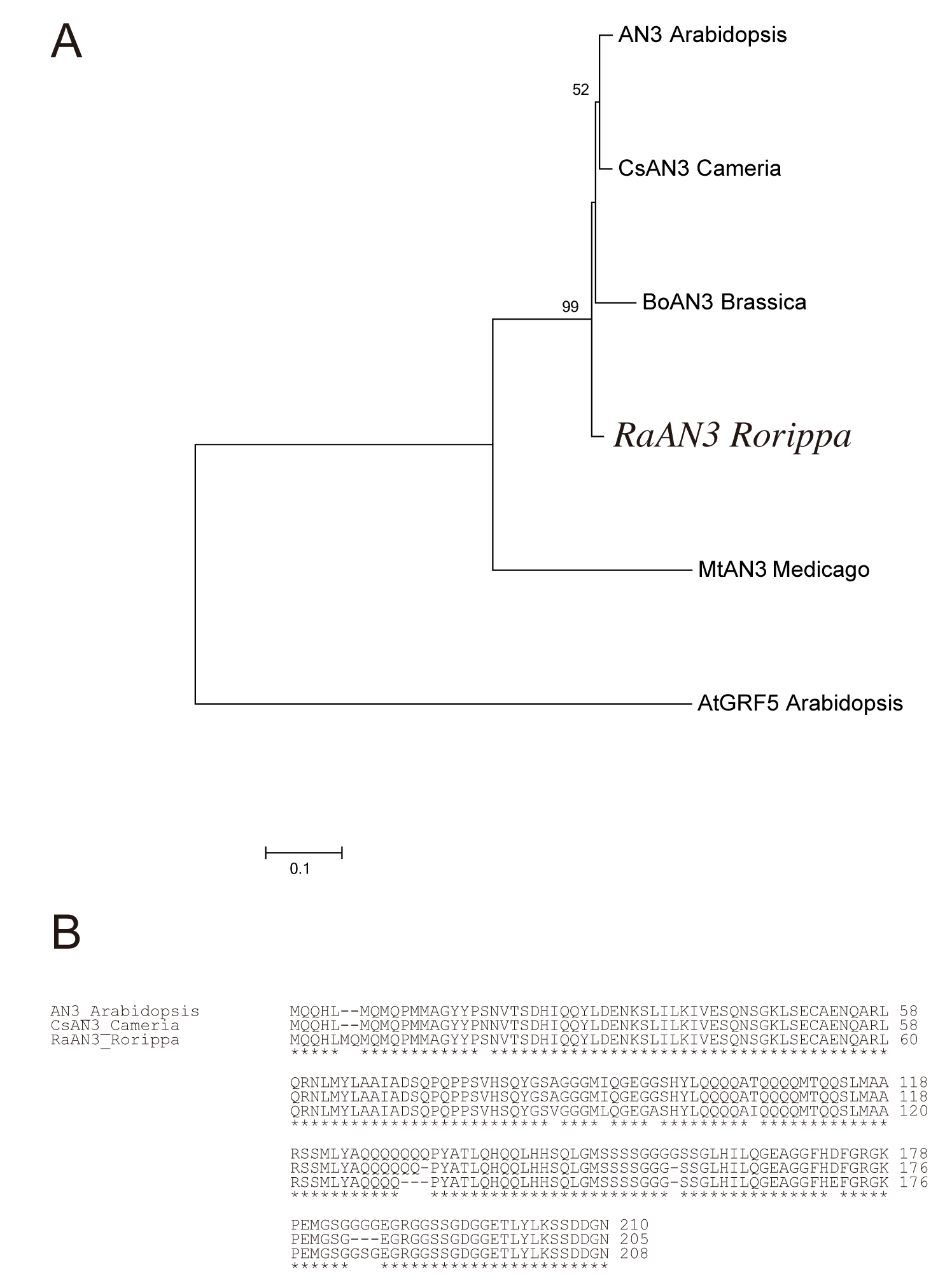

Supplement: S1 Fig — (TIF) [file pone.0141247.s001.tif]

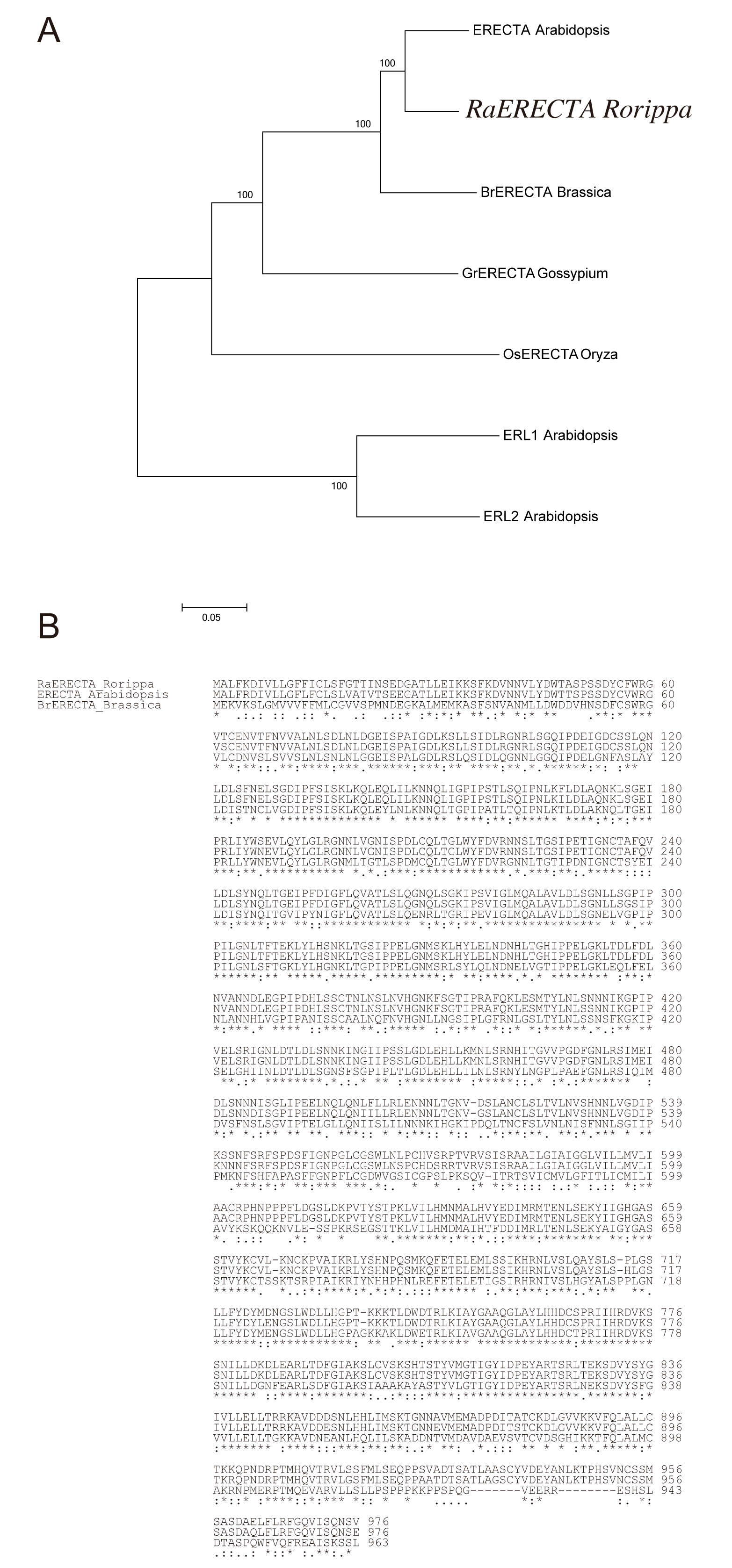

Supplement: S2 Fig — (TIF) [file pone.0141247.s002.tif]

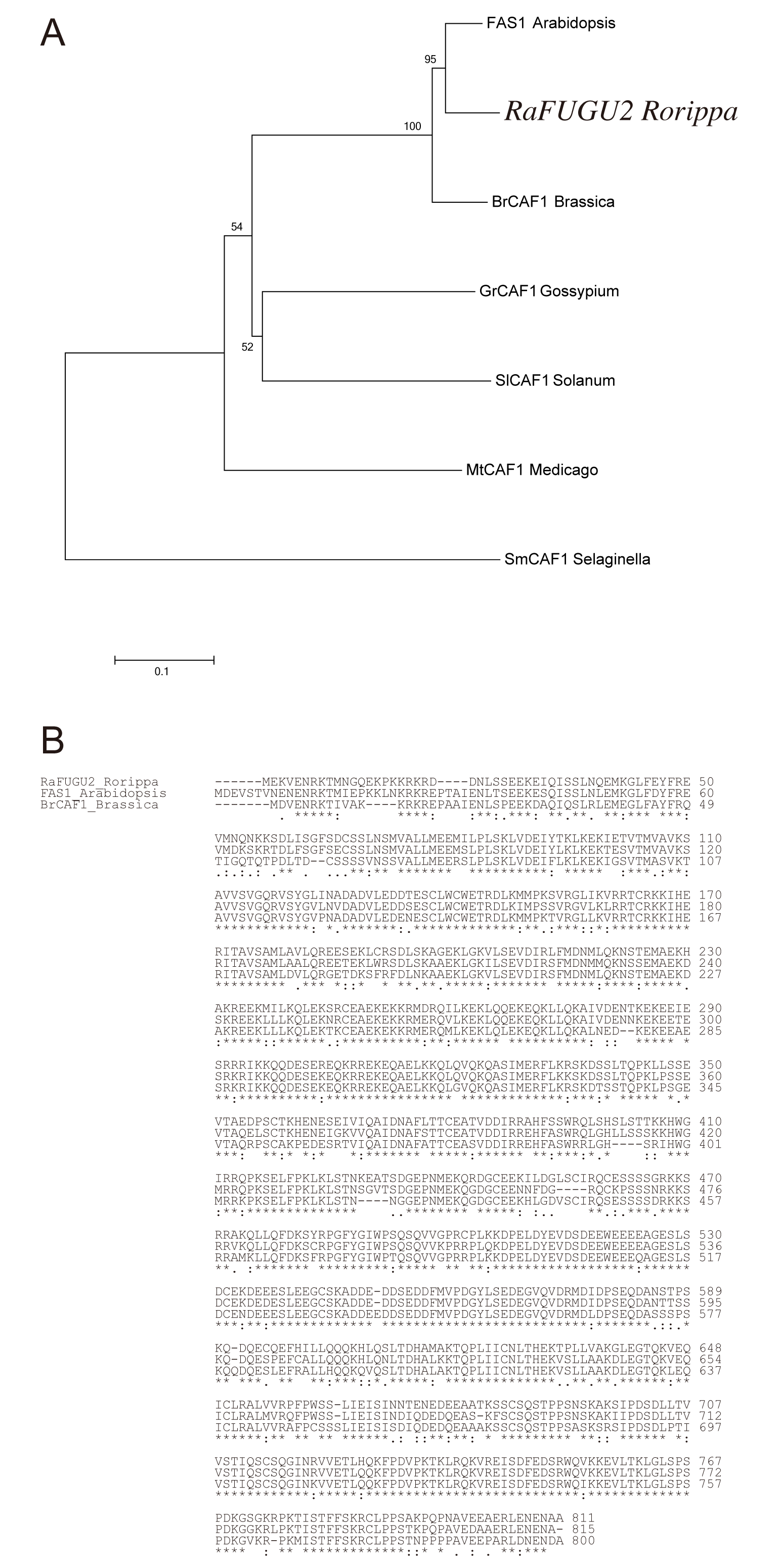

Supplement: S3 Fig — (TIF) [file pone.0141247.s003.tif]

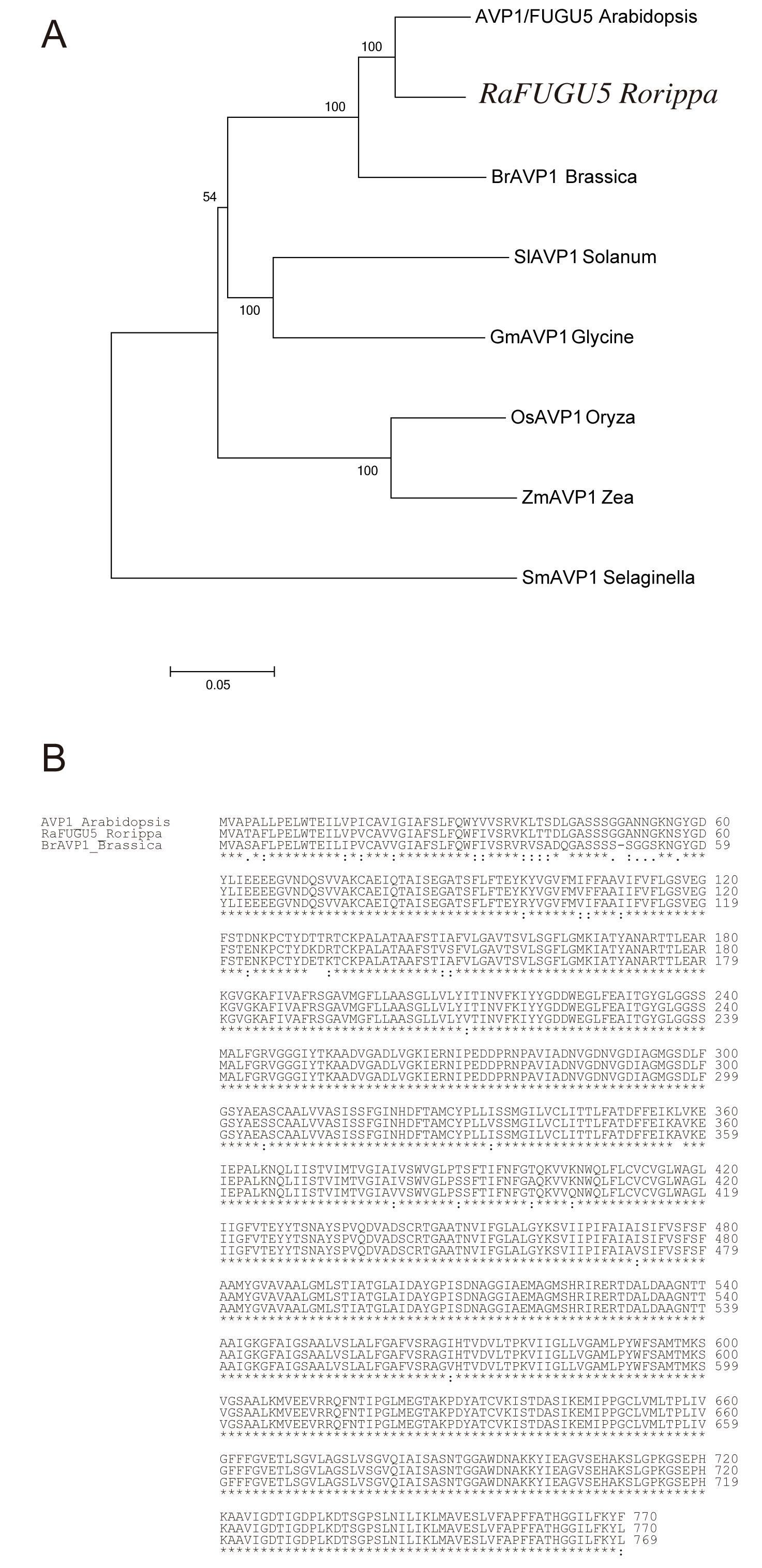

Supplement: S4 Fig — (TIF) [file pone.0141247.s004.tif]

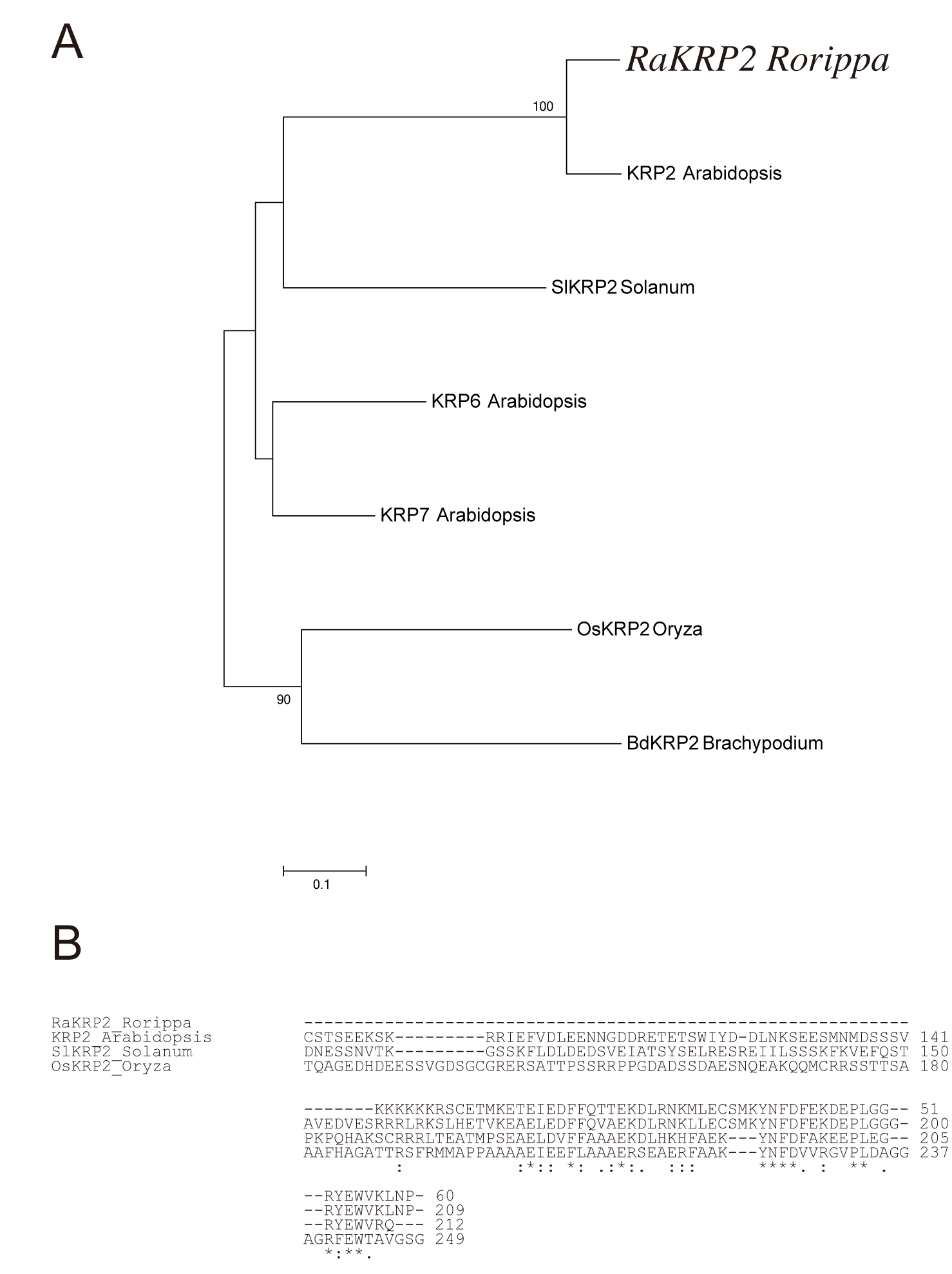

Supplement: S5 Fig — (TIF) [file pone.0141247.s005.tif]
